# Metabolomic and transcriptomic response to imatinib treatment of gastrointestinal stromal tumour in xenograft-bearing mice

**DOI:** 10.1016/j.tranon.2023.101632

**Published:** 2023-02-10

**Authors:** Szymon Macioszek, Danuta Dudzik, Rafał Bartoszewski, Tomasz Stokowy, Diether Lambrechts, Bram Boeckx, Agnieszka Wozniak, Patrick Schöffski, Michał J. Markuszewski

**Affiliations:** a Department of Biopharmaceutics and Pharmacodynamics, Medical University of Gdańsk, Hallera 107, 80-416 Gdańsk, Poland; b Department of Biophysics, Faculty of Biotechnology, University of Wrocław, F. Joliot-Curie 14a Street, 50-383 Wrocław, Poland; c IT Division, University of Bergen, 5021, Bergen, Norway; d Laboratory of Translational Genetics, KU Leuven and VIB Center for Cancer Biology, Leuven, Belgium; e Laboratory of Experimental Oncology, Department of Oncology, KU Leuven, and Department of General Medical Oncology, University Hospitals Leuven, Leuven Cancer Institute, Leuven, Belgium

**Keywords:** Metabolomics, Transcriptomics, Imatinib, GIST, purines, Multi-omics, GIST, gastrointestinal stromal tumour, GC-MS, gas chromatography coupled to mass spectrometry, TKI, tyrosine kinase inhibitor, ctDNA, circulating tumour DNA, QC, quality control, VIP, variable importance into projection, IT-GIST, imatinib-treated GIST, NT-GIST, non-treated GIST, OPLS-DA, orthogonal projection to latent structure- discriminant analysis, PCA, Principal Component Analysis, PDGFRA, Platelet Derived Growth Factor Receptor Alpha, NIST, National Institute of Standards and Technology, 3-OHB, beta-hydroxybutyrate, BSTFA, (N,O-bis(trimethylsilyl)trifluoroacetamide) with 1% TMCS (trimethylchlorosilane), FDR, false discovery rate, FKPM, fragments per kilobase of transcript per million fragments mapped

## Abstract

**Background:**

Although imatinib is a well-established first-line drug for treating a vast majority of gastrointestinal stromal tumours (GIST), GISTs acquire secondary resistance during therapy. Multi-omics approaches provide an integrated perspective to empower the development of personalised therapies through a better understanding of functional biology underlying the disease and molecular-driven selection of the best-targeted individualised therapy. In this study, we applied integrative metabolomic and transcriptomic analyses to elucidate tumour biochemical processes affected by imatinib treatment.

**Materials and methods:**

A GIST xenograft mouse model was used in the study, including 10 mice treated with imatinib and 10 non-treated controls. Metabolites in tumour extracts were analysed using gas chromatography coupled with mass spectrometry (GC-MS). RNA sequencing was also performed on the samples subset (n=6).

**Results:**

Metabolomic analysis revealed 21 differentiating metabolites, whereas next-generation RNA sequencing data analysis resulted in 531 differentially expressed genes. Imatinib significantly changed the profile of metabolites associated mainly with purine and pyrimidine metabolism, butanoate metabolism, as well as alanine, aspartate, and glutamate metabolism. The related changes in transcriptomic profiles included genes involved in kinase activity and immune responses, as well as supported its impact on the purine biosynthesis pathway.

**Conclusions:**

Our multi-omics study confirmed previously known pathways involved in imatinib anticancer activity as well as correlated imatinib-relevant downregulation of expression of purine biosynthesis pathway genes with the reduction of respectful metabolites. Furthermore, considering the importance of the purine biosynthesis pathway for cancer proliferation, we identified a potentially novel mechanism for the anti-tumour activity of imatinib. Based on the results, we hypothesise metabolic modulations aiming at the reduction in purine and pyrimidine pool may ensure higher imatinib efficacy or re-sensitise imatinib-resistant tumours.

## Highlights

• Imatinib treatment influences both the metabolome and transcriptome of GIST cell
• Changes in purine, pyrimidine, butanoate, and amino acid metabolism were detected
• Expression of kinase activity, immune response, and purine biosynthesis-related genes were altered
• Transcriptomic results support insights gained from the metabolomic study

## Introduction

Gastrointestinal stromal tumour (GIST) is a rare, yet the most common gastrointestinal tract sarcoma with a prevalence ranging between 1-10/100,000 population depending on a country [1], [2], [3], [4]. Approximately 85% of all cases are caused by mutations in *KIT* or *PDGFRA* (Platelet Derived Growth Factor Receptor Alpha) genes. These genes encode receptor tyrosine kinase proteins, which following activation by a ligand, promote cell growth and proliferation through such signalling pathways as the PI3K/AKT pathway, the STAT pathway, and the MAP kinase pathway. Mutation of *KIT* or *PDGFRA* leads to constitutive kinase activation independent of the ligand, and consequent unrestrained induction of signalling cascades leading to tumour proliferation at an abnormally high rate [5]. Before 2000s, due to the resistance to standard chemotherapy and radiation therapy, the only treatment option for localised tumour was its resection, which eventually led to disease recurrence [6]. Imatinib, introduced in 2001 as the first targeted anticancer drug, revolutionised GIST treatment. Imatinib specifically inhibits tyrosine kinase enzymes by direct binding to the KIT receptor. Therefore, the signal to constant proliferation is halted [7,8]. The GIST responsiveness to imatinib was observed to be related to the type of underlying mutation. A mutation in *KIT* exon 11 or in *PDGFRA* gene (except exon 18 p.D842V) is associated with a higher response rate and longer recurrence-free survival than GISTs with a *KIT* exon 9 mutation [9,10]. Moreover, wild-type GISTs (not related to *KIT* and *PDGFRA* mutations) are most likely to exhibit primary resistance to imatinib [11].

Despite initially promising therapeutic outcome in Tyrosine Kinase Inhibitor (TKI) sensitive tumours, 85–90% of patients experience disease progression within 20–24 months as the tumour develops resistance [12]. This phenomenon is related to secondary mutations in *KIT* or *PDGFRA* genes [13]. While there are a growing number of studies focusing on the types of mutation that develop during TKI therapy, little is known about downstream metabolic processes that occur in the tumour cells under the influence of the treatment.

Omics has launched the era of revolutionary molecular medicine including cancer research, which fundamentally shifts the strategy from single-target to global analysis and from hypothesis-driven to discovery-based research [14]. Omics sciences are aimed at describing a whole set of genes, messenger RNA molecules, proteins, or metabolites, in biological matrices. This holistic framework starts from genomic testing, which is now more commonly applied for the analysis of circulating tumour DNA (ctDNA) [15,16]. In a form of liquid biopsy, ctDNA can provide early diagnosis as well as insight into tumour biology and therapy resistance. Metabolites are the last in the omics cascade and thus, they are the closest to the cancer phenotype since the alteration of oncogenes influences enzyme activity, and consequently, metabolite concentrations.

Metabolic reprogramming is one of the cancer hallmarks. It refers to the phenomenon that cancer cells reprogram some of their metabolisms, largely driven by the unique chemical microenvironment in cancer tissues [17]. Likewise, in stress situations such as anticancer drug treatment, cancer metabolism is able to rewire to adapt to new conditions. Therefore, metabolomics-driven understanding of cancer phenotype promises a tool for overcoming resistance by targeting those metabolic alterations. Integrative studies may better expound the drug mechanism of action, and facilitate the development of new or validate current drug targets enabling bench to bedside translation.

**Fig. 1:**
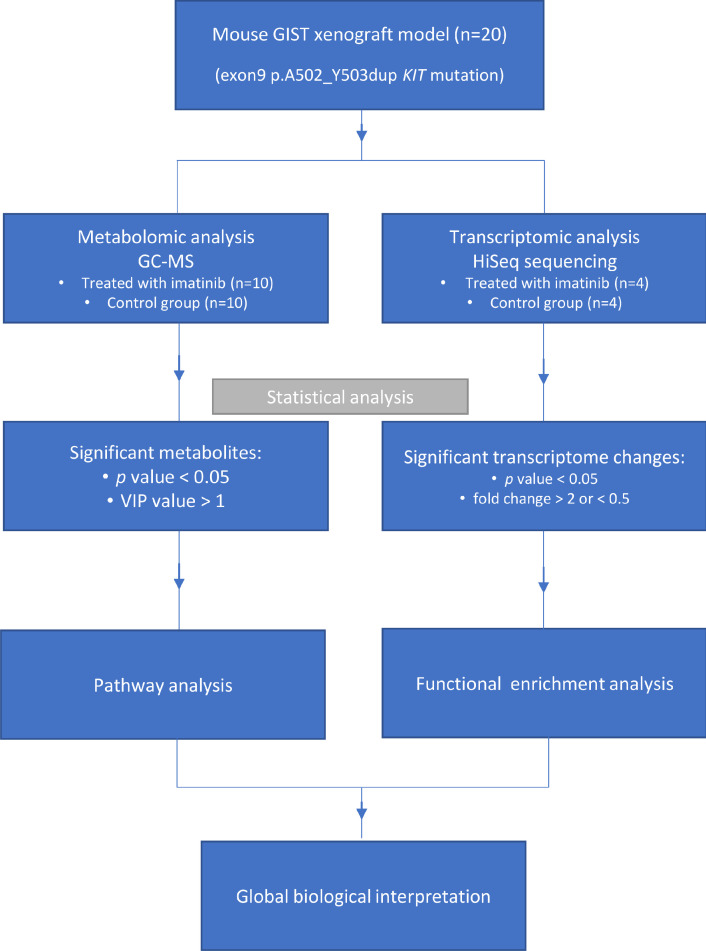
Flow chart illustrating the main steps of the study.

Both transcriptomics and metabolomics have been successfully applied in the studies of anticancer therapies on the tumour transcriptome and metabolome. For instance, thanks to the transcriptomic analysis, Thomas et al. discovered the degradation of the androgen receptor as a possible mechanism of a novel galeterone analogue in castration resistant prostate cancer [18]. In a study by Li et al, metabolomics was helpful to prove the anticancer activity of a Chinese herb-derived tricin against non-small cell lung cancer cells by underlining the role of sphingosine-1-phospahate [19]. Notably, the metabolomic consequences of imatinib treatment for GIST have not been studied, whereas there are only few reports of this drug's effect on gene expression profiles [20], [21], [22]. To the best of our knowledge, this is the first study to employ both metabolomics and transcriptomics in GIST tissue. Therefore, we applied metabolomic and integrative transcriptomic analyses of GIST tumour xenografts harvesting a *KIT* gene mutation, treated with imatinib and from non-treated controls. The results of this joined metabolomic and transcriptomic study confirmed previously known mediators and pathways involved in the anticancer activity of imatinib as well as identified purine metabolism as a novel target of this drug contributing to its anti-tumour activity. The general concept of the study is presented in the flow chart in Fig. 1.

## Materials and methods

### Study design and sample collection

GIST samples were collected from a patient-derived xenograft mouse model (UZLX-GIST2 passage 10), established and treated in the Laboratory of Experimental Oncology, KU Leuven, Belgium. The model establishment and sample collection were approved by the Ethics Committee, University Hospitals Leuven (S53483), and *in vivo* experiments were approved by the KU Leuven Ethics Committee for Animal Research (project number P175/2015). A xenograft model was established from a GIST fragment resected from a patient, which was subcutaneously transplanted into immunodeficient mice (*nu/nu* NMRI). When the tumour reached >1000mm^3^, it was re-transplanted into the next generation of mice. In this study, ten mice were treated orally with 50 mg/kg imatinib twice a day for three weeks. Another ten mice belonged to the control group, which received water instead. The ex-mouse tumour samples were left-over material, collected in the frame of previously performed *in vivo* experiment. The model retained its human origin and histopathological characteristic (KIT and DOG1 immunopositivity) as observed in the donor tumour (Fig. S1). Furthermore, all ex-mouse samples carried a mutation in *KIT* (exon9 p.A502_Y503dup), which is responsible for dose-dependent resistance to imatinib treatment.

### Sample preparation

Samples were snap frozen at the end of the experiment and stored at -80°C prior to analysis. The tumours were immersed in liquid nitrogen, cut into 50-70 mg sections, and transferred to Eppendorf® tubes with 2 mm diameter zirconium oxide homogenisation beads. Ten microlitres of a methanol and water mixture (1:1, *v/v*) was added for each mg of the tumour. The homogenisation was carried out in a Bullet Blender homogeniser (Next Advance, USA) (speed 10) in two 5-min cycles, between which the samples were cooled on ice. Then, 100 µL of each homogenate was subjected to further preparation for the GC-MS analysis. The homogenate fractions were mixed with 200 µL of cold methanol containing 1 mg/mL pentadecanoic acid (internal standard), centrifuged at 4000 × *g* at 4°C for 15 min, and 200 µL of the supernatant was evaporated in a MiVac DUO concentrator (GeneVac, UK). Then, the derivatization process was performed to convert metabolites into their volatile derivatives. First, 20 µL of O-methoxyamine HCl (15 mg/mL) in pyridine was added, and the samples were ultrasonicated and incubated for 16 h in a dark place at room temperature. Next, 20 µL N,O-bis(trimethylsilyl)trifluoroacetamide (BSTFA) with 1% trimethylchlorosilane (TMCS) was added, and the samples were placed in an oven (1h, 70⁰C for silylation). The final step included the addition of 100 µL of heptane to each sample.

Quality control (QC) samples were prepared simultaneously with the preparation of GIST samples. Fifteen microlitres of each homogenate were first collected to prepare a pooled QC sample following the same extraction procedure as applied for the experimental samples.

### GC-MS metabolomic profiling

GC-MS analysis was performed using a 7890B Agilent Technologies GC system with 7000 triple quadrupole (Agilent Technologies, United States). The analytical batch started with two heptane blanks, an extraction blank, and n-alkane standard solution (C10 – C40), followed by injections of QCs for system equilibration. Then, the GIST samples were injected in blocks in a randomised order, separated by the injection of a QC sample at constant intervals. Next, 1 µL of each sample was injected in a splitless mode into a ZB-5MS column (30 m length, 0.25 mm i.d., 0.25 µm film 95% dimethyl/5% diphenylpolysiloxane) from Phenomenex (United States). The helium carrier gas flow rate was set at 1 mL/min, and the injector temperature was at 250°C. The temperature gradient started at 60°C for 1 min and increased to 320°C at a rate of 8°C/min rate. The total time of analysis was 37.5 min. The detector transfer line, filament source, and quadrupole temperatures were set at 320°C, 230°C, and 150°C, respectively. The electron impact ionisation source voltage was set at 70 eV. The data were collected in scan mode over a mass range of 50−600 *m/z* at 2 spectra/s.

### Metabolomic data processing and analysis

Spectral deconvolution was carried out using Agilent Unknown Analysis software (Ver. B.09.00, Agilent Technologies) with NIST (National Institute of Standards and Technology) mass spectra library (Ver. 2014). Metabolites were identified based on mass spectral similarity and comparison of retention indices, calculated from the retention times of n-alkanes. The extracted peaks were aligned across all samples in Mass Profiler Professional ver. B.02.1 (Agilent Technologies) software. Thereafter, the entire batch pre-processing, assignment of the target ion and the qualifiers, and manual inspection of the acquired data including peak area and RT integration were performed in Agilent MassHunter Quantitative Analysis (Ver. B.09.00, Agilent Technologies). Peaks corresponding to the internal standard were inspected to verify the reproducibility of the derivatization procedure and GC-MS analysis. Finally, metabolites with a coefficient of signal variation higher than 30% in the QC samples were filtered out prior to statistical analysis.

Metabolomic data were subjected to univariate and multivariate statistical analysis in MATLAB 2016b software (MathWorks, Natick, MA, USA). Univariate methods included Student's t-test or U Mann-Whitney test (*p* value < 0.05) depending on data normality, while the multivariate algorithm was variable importance into projection (VIP value > 1) calculated from the orthogonal projection to latent structure discriminant analysis (OPLS-DA) model. Metabolites which were significant in both univariate and multivariate methods were considered in biological interpretation as compounds with the highest discriminating power between imatinib-treated (IT-GIST) and non-treated (NT-GIST) control tumours.

### RNA isolation and sequencing

Transcriptomic analysis was performed on eight GIST samples from integrative metabolomic study, among which four tumours were treated with imatinib and four belonged to the control group. RNA isolation and sequencing followed the procedure described by Nassar et al. [23]. GIST sections were incubated overnight in trypsin-EDTA at 4°C. Afterwards, trypsin was neutralised, and the tumour was minced and filtered through 70 μM and 40-μM pores (BD Biosciences). Single-cell suspensions of approximately 2 × 10^5^ cells were subjected to RNA extraction using an RNeasy Micro Kit (Qiagen). Ilumina TruSeq RNA Sample Preparation v2 was used for library generation, and sequencing was performed on a HiSeq4000 (Illumina). Reads were assigned to Ensembl IDs with the HTSeq software tool [24].

### Transcriptomic data analysis

The average number of useful reads per sample was 28,992,777. Raw count data were normalised using the fragments per kilobase of transcript per million fragments mapped method (FKPM). Only genes with a median expression level > 7 in at least one of the sample groups were considered for statistical analysis. For data overview, a cluster dendrogram was generated in R (ver. 4.0.2). Fold change computation, statistical analysis, and annotation were performed in Microsoft Excel and R programming environment (ver. 4.0.2) using the R Studio (ver. 1.4.1106). Statistically significant genes from the comparative analysis (fold change > 2 or < 0.5 and *p* value < 0.05 in Student's t-test) were subjected to functional enrichment analysis using online tools: EnrichR (https://maayanlab.cloud/Enrichr), Webgestalt (http://www.webgestalt.org), and MetaboAnalyst 5.0 (https://www.metaboanalyst.ca) [25].

Among the eight tumour samples analysed by RNA sequencing, two outliers were found with a much lower alignment of reads in the loci of coding genes (46-47% compared to 61-65% in other samples). The outliers are shown in the hierarchical clustering graph (Fig. S2). Therefore, during further analysis three versus three samples were eventually compared.

## Results

### GC-MS metabolomic analysis

**Fig. 2:**
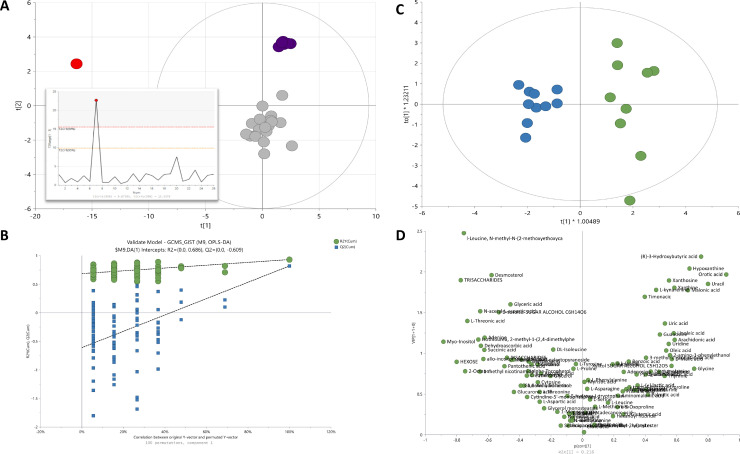
Multivariate statistical analysis of metabolomic data from imatinib-treated (n=10) and non-treated (n=10) GIST samples. (A) Clustering of QCs on a PCA score plot built on data acquired during GC-MS analysis of GIST tissue extracts. The grey dots represent the experimental samples, and the purple dots correspond to quality controls (QCs). The red dot indicates a detected outlier. Panel A also shows Hotelling's T2 plot with one critical outlier sample (T2Crit 99%). (B) OPLS-DA scatter score plot, R2=0.88, Q2=0.80, CV-ANOVA p-value 5.35 E-05. (C) Permutation test, the number of permutations 100; intercepts R2=0.0, 0.69, Q2=0.0, -0.466. Green colour represents non-treated controls while blue corresponds to imatinib-treated samples. (D) Volcano plot for the OPLS-DA model. Metabolites with highest VIP values and highest negative or positive pcorr highlight the metabolomic signature of GIST treatment.

GC-MS metabolomic analysis of tumour extracts identified 98 metabolites. An exemplary chromatogram of a QC sample is shown in Fig. S3. The quality of the analytical procedure was verified based on the Principal Component Analysis (PCA) analysis. The tight clustering of the QC samples proved the reproducibility of the analytical conditions throughout the acquired sequence (Fig. 2A). One outlying observation (non-treated sample) was detected by Hotelling´s T2 Range Plot based on PCA-X model and removed from further calculations.

### The comparison of metabolic profile of imatinib-treated and non-treated GIST

The signals of 98 detected metabolites were compared between IT-GIST and NT-GIST. The OPLS-DA supervised multivariate analysis indicated the discrimination between metabolic profiles of treated and non-treated tumours (Fig. 2B). For validation of the OPLS-DA model, CV ANOVA was calculated and the permutation test with 100 permutations showed that the model is valid (Fig. 2C). Variables contributed to the group separation were selected following the established multivariate VIP (>1.0) and p(corr) (> 0.05 or < 0.05) criteria. Independent univariate analysis based on *t*-test and false discovery rate FDR-adjusted p-value were performed. Twenty one metabolites were statistically significant according to both univariate and multivariate analysis. They included purines, pyrimidines, carbohydrates, organic acids, fatty acids, amino acids and derivatives. Significant metabolites are presented in Table 1 along with *p* value, FDR, VIP, and p(corr) values.

**Table 1:** Metabolites discriminating between imatinib-treated GIST (IT) (n=10) and non-treated controls (NT) (n = 10). Only statistically significant metabolites are presented. VIP – variable importance in projection. p (corr) - loadings scaled as a correlation coefficient (ranging from −1.0 to 1.0) between the model and original data. * - metabolites that did not pass FDR threshold of 0.05, however were significant in multivariate analysis.

Compound	Metabolite class	Percentage change (IT vs NT)	*p*-value	FDR	VIP	p(corr)
N-acetyl-L-aspartic acid	Amino acids and derivatives	+62%	0.00717	0.03533	1.2	-0.5
Malonic acid	Organic acids	-56%	0.01099	0.04669	2.0	0.8
(R)-3-Hydroxybutyric acid	Organic acids	-65%	0.00081	0.01378	2.5	0.8
Succinic acid	Organic acids	+44%	0.00509	0.03214	1.1	-0.6
D-Malic acid	Organic acids	-23%	0.02022	0.07486*	1.1	0.7
Linoleic acid	Fatty Acyls	-37%	0.00762	0.03533	1.5	0.7
Oleic acid	Fatty Acyls	-30%	0.01316	0.05163*	1.2	0.6
Arachidonic acid	Fatty Acyls	-40%	0.00711	0.03533	1.3	0.6
Hypoxanthine	Purines and purine derivatives	-69%	0.00659	0.03533	2.3	0.8
Adenine	Purines and purine derivatives	+53%	0.01143	0.04821	1.2	-0.6
Xanthine	Purines and purine derivatives	-63%	0.01182	0.04821	2.0	0.7
Uric acid	Purines and purine derivatives	-49%	0.00644	0.03533	1.5	0.6
Xanthosine	Purines and purine derivatives	-69%	0.01543	0.05621*	2.1	0.7
Uracil	Pyrimidines and pyrimidine derivatives	-59%	0.00004	0.00115	2.1	0.9
Orotic acid	Pyrimidines and pyrimidine derivatives	-57%	0.00002	0.00110	2.0	0.9
Uridine	Pyrimidines and pyrimidine derivatives	-34%	0.01129	0.04821	1.3	0.6
Desmosterol	Steroids and steroid derivatives	+143%	0.02202	0.07245*	2.2	-0.7
Dehydroascorbic acid	Lactones	+45%	0.00285	0.02420	1.1	-0.6
L-Threonic acid	Carbohydrates	+13%	0.00004	0.00115	1.5	-0.7
Allo-inositol	Carbohydrates	+27%	0.00552	0.03214	1.0	-0.6
Myo-Inositol	Carbohydrates	+34%	1.01E-06	0.00010	1.2	-0.9
Hexoses	Carbohydrates	+22%	0.00011	0.00230	1.0	-0.8

### Imatinib treatment and altered gene expression

Differential expression analysis of IT-GIST and NT-GIST identified a large number of statistically significant transcriptome changes (by at least 2 fold change and with a *p* value below 0.05) that included 167 upregulated and 364 downregulated genes (Table S2).

**Fig. 3:**
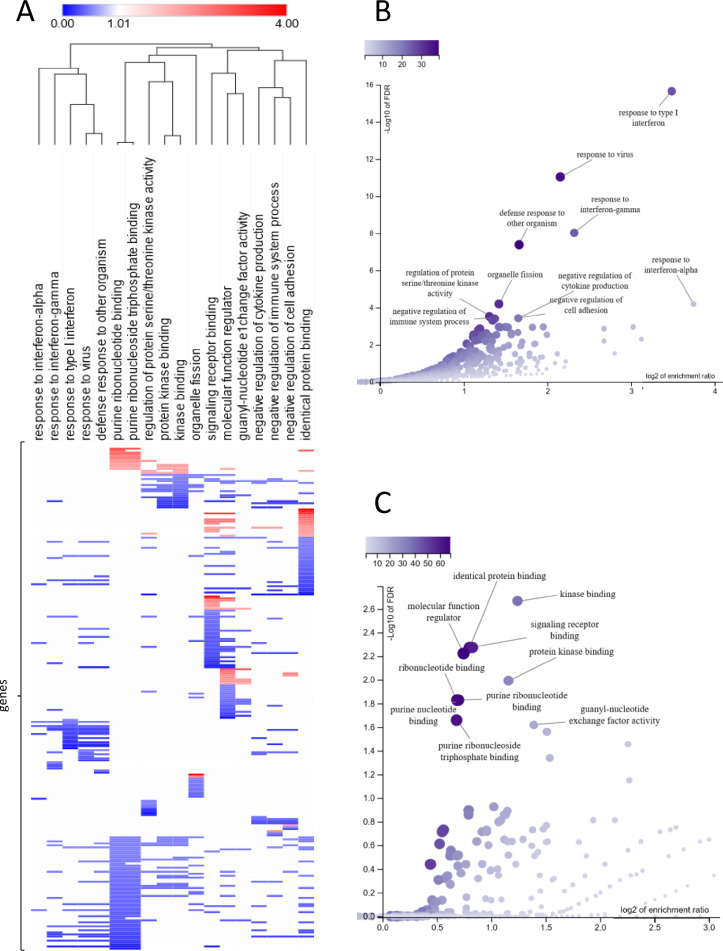
Imatinib-related effects on biological processes. (A) and molecular functions (B) based on the gene ontology assignment of transcriptomic changes. The mRNA levels in IT-GIST that were changed significantly (*p* value 0.05) by at least 2 folds were assigned with the WebGestalt database to cellular signalling pathways and selected based on FDR ˂ 0.05. (C) The changes in the expression of genes assigned to the identified signalling pathways are presented as the hierarchically clustered heatmap. The heat map generation and hierarchical clustering were performed with the Morpheus web server (https://software.broadinstitute.org/morpheus/). The assigned gene expression changes are provided in Table S1.

The follow-up functional assignment of imatinib-modulated genes was performed with Enrichr [26] and WEB-based Gene SeT AnaLysis Toolkit (WebGestalt) [27], using strict criteria (*p* value *p* < 0.05 and *q* value *q* < 0.05). As shown in Fig. 3, the expression profile specific for IT-GIST corresponded well with changes related to protein kinase activity, purine metabolism, modulation of immune responses, and cytokine and interferon signalling. The reduction in the expression of genes involved in kinase-related signalling as the most affected pathway is in good agreement with imatinib mechanism of action being the KIT inhibition.

## Discussion

The complexity of cancer is still beyond our understanding. The insights into molecular mechanisms and interactions determining the relationship between disease and response to the pharmacotherapy are crucial for the development of both effective, molecularly targeted personalised therapies with reduced cytotoxicity and adverse effects as well as reliable biomarkers. Such biomarkers could serve both for early disease prediction and to monitor treatment efficacy. The recent advances in omics technologies and especially integrative multi-omics methods, offer a holistic view that serves to obtain a full cellular readout and accelerates research to pave the way for better understanding of disease aetiology, treatment and prevention. Although both metabolomic or transcriptomic analyses have been commonly applied in cancer research [28], [29], [30], [31], their simultaneous application is challenging and thus underrepresented in literature [32]. Nevertheless, integrating these omics could provide new insights into the drug impact on the disease-related mechanism. The point addressed in this study was to implement parallel metabolomic and transcriptomic approaches to analyse the relationship between GIST and imatinib. Although imatinib is the standard first-line treatment for advanced, inoperable, and metastatic patients with a vast majority of GIST molecular subtypes [33], tumours acquire secondary resistance during the ongoing therapy. Notably, imatinib can only bind to active conformations of KIT, and both primary and secondary resistance to imatinib can be partially explained by a conformational shift in the kinase domain of KIT that favours the activated state [34,35]. The conformational changes in KIT due to exon 11 mutations disrupt the autoinhibitory domain of the receptor and permit continuous kinase activation [36]. Hence, imatinib can bind to inactive mutated KIT and efficiently prevent from its continuous activation upon ligand occurrence. Mutations in exon 9 are believed to mimic the conformational change that the extracellular KIT receptor undergoes when ligand is bound, thus leading to dimerization and constitutive activation [37]. Hence, the mutated KIT is in active-like conformation that prevents the efficient imatinib binding. Despite new generations of TKIs exhibiting clinical benefits in imatinib-resistant patients, none of them surpasses the efficacy of imatinib. Hence, the observation of metabolomic and transcriptomic changes occurring during imatinib treatment could be the starting point to elucidate the molecular background of tumour response and resistance.

Since numerous reports have shown that patient-derived specific mouse xenografts provide valid tools for testing anticancer therapy and related drug response [38,39], we used this model in our study. Furthermore, patient-derived xenograft models have already been well-established for GIST drug candidates testing, including imatinib [40,41]. Although it is possible to perform data analysis based on results from similar but unrelated experiments [42], [43], [44], in our studies to ensure unbiased integration of transcriptomic and metabolomics data, the same samples were analysed simultaneously with both of these omic approaches.

**Fig. 4:**
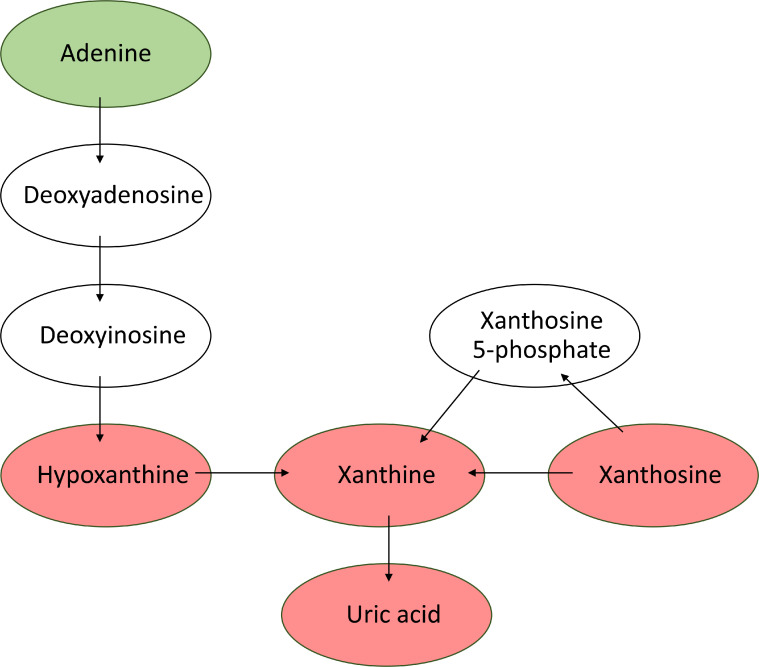
The model of imatinib effects on the purine pathway. Red boxes represent downregulated metabolites, while green colour indicates upregulation.

Our analyses identified that treatment of tumours with imatinib affects the metabolomic profile of compounds associated mainly with purine and pyrimidine metabolism, butanoate metabolism, as well as alanine, aspartate, and glutamate metabolism. We noticed decreased levels of purine metabolism components: hypoxanthine, xanthine, xanthosine, and uric acid (depicted in Fig. 4). Purines play critical roles in cells as they constitute the building blocks of DNA and are a source of energy [45,46]. The amount of purines in a cell is maintained mainly by the salvage pathway, in which the degraded bases and nucleosides are recycled by adenine phosphoribosyltransferase or hypoxanthine-guanine phosphoribosyltransferase. However, in cancer, the demand for purines is higher and *de novo* biosynthesis must be elevated [47]. Without sufficient production of purines, tumour cells are not able to proliferate. Hence, inhibition of purine biosynthesis can presumably constitute a part of anticancer activity resulting from imatinib administration. Furthermore, a significant increase in adenine was also observed in GIST tissue after imatinib treatment. Importantly, adenine has been shown to strongly inhibit the growth of cancers along with the rate of tumour cells proliferation. The proposed mechanism of anti-tumour adenine activity includes activation of the AMPK/mTOR signalling pathway, which induces autophagic cell death [48,49]. Taken together, the observed changes in all purine pathway components are in good agreement with imatinib anticancer activity.

Similarly to purine metabolism, the pyrimidine pathway is also disturbed in IT-GIST. Uridine and orotic acid, which are required for cell growth as intermediates of pyrimidine and RNA synthesis [50,51], were both reduced in the treated tumour. Hence, the reduced availability of these metabolites in cancer cells may reflect imatinib treatment-related inhibition of cancer cells growth.

We have also observed significant reduction in members of unsaturated fatty acids biosynthesis pathway: linoleic acid, oleic acid, and arachidonic acid. Similar, imatinib-related reduction in linoleic acid was previously reported by Jiye A et al. [52]. Cancer cells are characterized by deregulation of fatty acids uptake and metabolism, as well as reactivation of their de novo synthesis that supports cancer progression and survival [53]. Notably, unsaturated fatty acids contribute to increased membrane fluidity, which can support cell adhesion, migration, and metastatic potential [53,54]. However, cancer cells that display low membrane fluidity are resistant to chemotherapeutics because of reduced drug uptake [55]. Hence imatinib-related reduction in unsaturated fatty acid levels can be considered both a therapeutic effect or a drug resistance mechanism. Answering this question requires further studies.

We also noted a significant reduction in beta-hydroxybutyrate (3-OHB), being the main physiological ketone body. However, depending on the tumour's energetic phenotype, changes in 3-OHB levels can be attributed to both cancer progression and suppression [56]. “Oxidative cells” would use 3-OHB as an additional energy source to increase their growth when this metabolite is available. On the other hand, other cells with a more “glycolytic, Warburg-like phenotype” would be unable to metabolise 3-OHB. It could accumulate intracellularly and inhibit tumour growth via signalling and epigenetic mechanisms (Bartmann et al. 2018). Hence, the imatinib-related consequences of this metabolite reduction should be carefully considered.

Finally, we have observed alterations in the alanine, aspartate, and glutamate metabolism pathways. Tumours silence the expression of genes required for the synthesis of some amino acids, producing auxotrophy, whereas an increase in the synthesis of other amino acids can be used by cancer cells to preserve redox homoeostasis, maintain α-ketoglutarate concentrations, or for the import of other amino acids [57]. Hence, imatinib-related changes in these pathways could be either a drug resistance mechanism or a consequence of its therapeutic effect, and further studies are required to elucidate which of these answers is correct.

Next, to follow the imatinib-related changes in the transcriptome, we performed parallel next-generation sequencing profiling of ex-mouse tumours treated with this drug. The treatment resulted in meaningful changes in the expression of the genes involved in kinase activity, confirming the mechanism of action of imatinib [58]. Furthermore, we have also observed a significant reduction in transcripts related to immune responses, including virus defence and cytokine production. Interestingly, imatinib has been previously reported to suppress cytokine production [59,60] as well as cytokine-dependent imatinib resistance has been described [61]. Our observation is further supported by the findings of Wolf et al., who showed that imatinib inhibits TNF-α production, and thus has anti-inflammatory effects [62]. Finally, in previous work, Liu et al. reported that KIT signalling contributes to the type I interferon pathway, whereas KIT inhibition attenuates tumour immunogenicity. In their mouse model, inhibition of oncogenic KIT in GIST reduced type I interferon production [63]. Interestingly, although a recent study excluded imatinib use as an anti SARS-Cov-2 drug [64], this compound has been shown to reduce the expression of genes with “proviral” functions, including Carcinoembryonic Antigen-Related Cell Adhesion Molecule 1 gene (*CEAMCAM1*) [65]. Similar results were observed in our analysis.

Furthermore, our transcriptomic analysis revealed the influence of imatinib on purine and pyrimidine metabolism, including the regulation of ribonuclease activity and purine ribonucleoside triphosphate binding. These findings are in perfect agreement with the results of the metabolomic analysis, where we observed reduction in purine and pyrimidine pathway metabolites. Hence, the imatinib-related downregulation of the expression of purine biosynthesis pathway-related genes may lead to the observed reduction in respectful metabolites. Considering the importance of the purine biosynthesis pathway for cancer proliferation, these data identify a novel mechanism contributing to the antitumor activity of imatinib.

We believe our findings can be an impulse for another kind of cancer phenotype modulation having clinical relevance. In terms of GIST therapy, interesting observations regarding intervention in cancer metabolism were made for glutaminase inhibitors, which were already the object of a clinical trial in GIST (NCT02071862). A study by Lee et al. revealed glutamine metabolism as crucial for sarcomagenesis due to the fact that glutamine generates TCA cycle intermediates [66]. A remarkable role of TCA cycle in tumour response to imatinib was also indicated in our results by statistically significant changes in malonate, 3-hydroxybutyrate, succinate, and malate. Furthermore, glutamine participates in aspartate production, which constitutes a major carbon source for purine and pyrimidine synthesis. Therefore, it is possible that modulation of glutamine or aspartate metabolism may have an effect in purine and pyrimidine pathways, which were significantly affected during imatinib treatment in our study. Vitiello et al. also proposed metabolic targeting can enhance the anti-tumour effects of imatinib by increasing glycolytic activity through mitochondrial inhibition [67]. This is in agreement with the numerous studies reporting an association between imatinib resistance and increased rates of glycolysis [68], [69], [70], [71], [72]. Furthermore, a recent report showed that the inhibition of glucose transporter GLUT1, the first rate-limiting factor in the glucose metabolic pathway, overcomes imatinib resistance [73]. Notably, an enhanced glycolysis and related disruptions in mitochondrial metabolism are common functional markers of many TKIs-resistant cells [74]. Therefore, despite the fact that resistance to TKIs is often a consequence of kinase mutations, it is also associated with oxidative stress responses, hypoxia signatures, and apparent metabolic reprogramming of the cells [74]. The enhanced glycolysis protects TKIs-resistant cancer cells from reactive oxygen species accumulation and oxidative stress related cell death [75,76]. Hence, the supporting therapies that will prevent this increased metabolic plasticity of cancer cells could provide an effective solution to overcome TKIs-resistance. We believe targeting cancer metabolism may be a key to re-sensitisation of drug resistant GIST; however, the multifactorial dependencies of gene expression, proteins, and metabolites make it difficult to predict the eventual cancer phenotype. Here, based on our results, we hypothesise further research is highly promising to investigate the modulation of purine and pyrimidine metabolism for augmenting imatinib efficacy.

Moreover, the results of this study can also drive development of new prognostic risk models in GIST. Present models assume that disease-free survival is correlated with tumour location, size, mitotic index, or mutational status [33,77]. As the current risk classification is still insufficient [78], new prospective studies are required to assess the prognostic value of metabolic biomarkers. Our study confirms the important role of specific metabolites in tumour treatment response; hence, their potential to predict the therapy outcome and patient survival should be further verified.

Taken together, our joined metabolomic and transcriptomic study not only confirmed previously known mediators and pathways involved in imatinib anticancer activity but also identified novel targets of this drug, including deregulation of fatty acid synthesis and purine metabolism. Our observations derive from the GIST xenografts grown in mice; therefore, the study should be further reproduced in human clinical samples.

## Funding

This work was supported by the 10.13039/501100004442National Science Centre Poland [grant Preludium no 2018/29/N/NZ7/02908] and by the project POWR.03.02.00-00-I026/17-00 co-financed by the European Union through the European Social Fund under the Operational Programme Knowledge Education Development 2014–2020.

## CRediT authorship contribution statement

**Szymon Macioszek:** Conceptualization, Investigation, Formal analysis, Writing – original draft. **Danuta Dudzik:** Formal analysis, Data curation, Visualization, Writing – review & editing. **Rafał Bartoszewski:** Formal analysis, Writing – original draft. **Tomasz Stokowy:** Software, Data curation. **Diether Lambrechts:** Investigation, Data curation. **Bram Boeckx:** Software, Data curation. **Agnieszka Wozniak:** Conceptualization, Resources, Writing – review & editing. **Patrick Schöffski:** Supervision, Writing – review & editing. **Michał J. Markuszewski:** Conceptualization, Supervision, Writing – review & editing.

## Declaration of competing interest

The authors declare no conflict of interest associated with this publication.

## Appendix Supplementary materials

Image, application 1
