# Supplementary material for: Metabolomic and transcriptomic response to imatinib treatment of gastrointestinal stromal tumour in xenograft-bearing mice
Source: Transl Oncol. 2023 Feb 10;30:101632. doi: 10.1016/j.tranon.2023.101632 (PMC9945753; doi:10.1016/j.tranon.2023.101632)
Supplement: Supplementary file 1 [file mmc1.docx]

Figure S1. Histological characterisation of the ULZX-GIST2 patient-derived xenograft model of GIST. The same morphology by hematoxylin and eosin (H&E) staining and presence of GIST specific markers (KIT and DOG1 – Discovered on GIST 1) by immunohistochemistry were observed in an early passage (p.1) and corresponding passage (p.10) used for the current analysis.


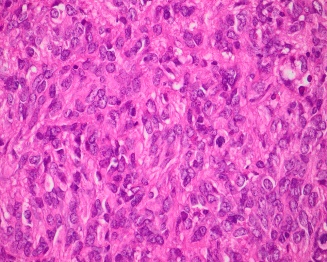

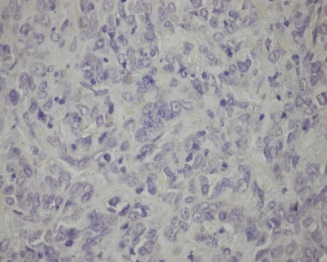

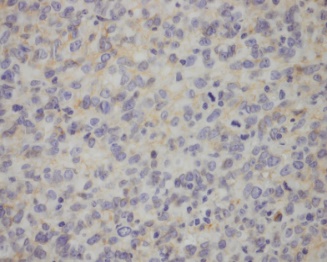

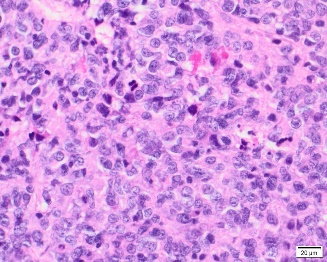

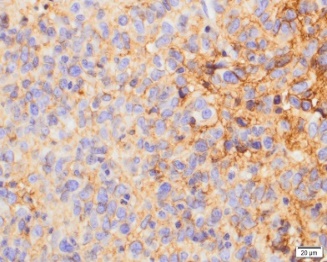

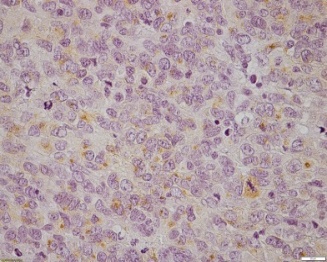


**400x**

H&E

KIT

DOG1

**UZLX-GIST2**

passage 1

passage 10


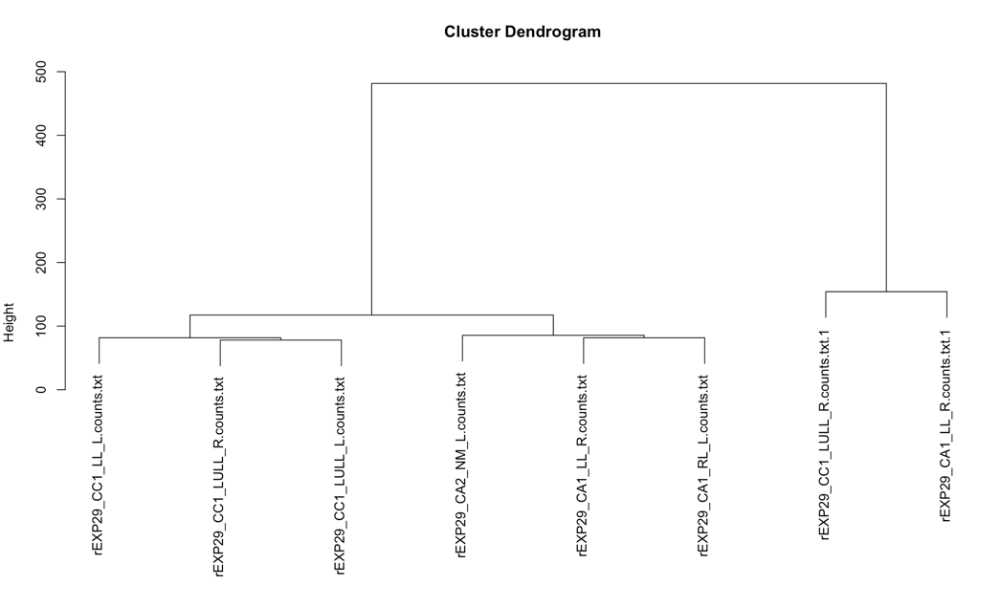


Figure S2. Cluster dendrogram showing similarities between the analysed transcriptomes. On the right, two outliers can be noticed. The remaining six samples are grouped into two clusters according to applied imatinib treatment.


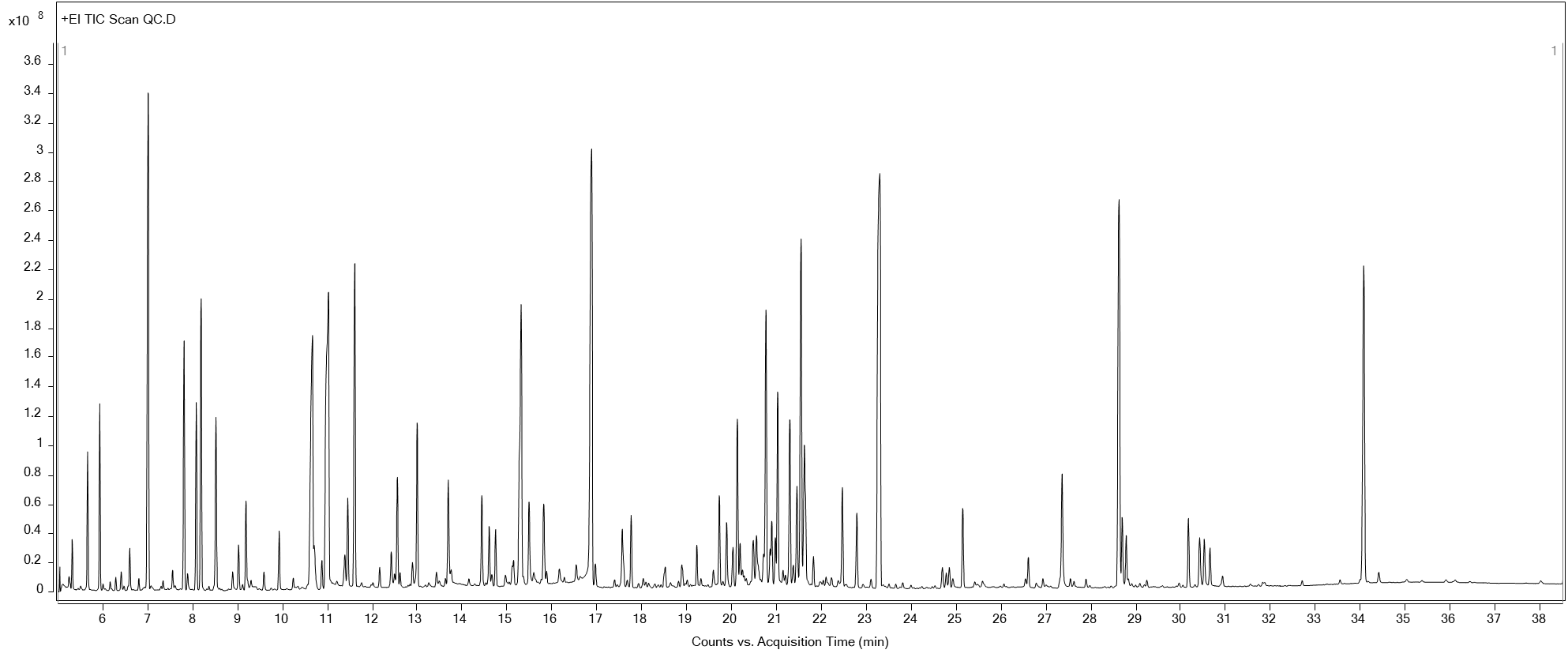


Figure S3. Exemplary chromatogram of a GIST tissue extract obtained during GC-MS analysis.

Table S1. Enrichment analysis results showing significantly affected Biological Processes (A) and Molecular Functions (B), performed using Webgestalt tool (http://www.webgestalt.org). The table includes genes contributing to particular processes or functions.

A)

| **GO symbol** | **Biological Process** | **Size** | **Overlap** | **pValue** | **FDR** | **Genes** |
| --- | --- | --- | --- | --- | --- | --- |
| GO:0034340 | response to type I interferon | 89 | 26 | 0 | 0 | EGR1; IFI27; IFI6; OAS1; SAMHD1; IRF1; IRF7; OAS3; MX1; USP18; IFIT3; WNT5A; OASL; OAS2; ISG15; XAF1; IFITM1; MX2; ZBP1; STAT2; RSAD2; IFI35; IFIT1; IFIT2; ISG20; IFITM2 |
| GO:0009615 | response to virus | 319 | 37 | 2.13E-14 | 9.06E-12 | PMAIP1; IFI27; IFI6; OAS1; SAMHD1; IRF1; IRF7; OAS3; TLR3; MX1; IFIT3; GBP1; OASL; OAS2; IFIT5; ISG15; IFITM1; IFIH1; DHX58; DDX58; MX2; STAT2; RSAD2; IFI44L; IFIT1; CXCL10; RTP4; NT5C3A; BCL3; IFIT2; LGALS9; GATA3; UNC93B1; ISG20; IFI44; GBP3; IFITM2 |
| GO:0034341 | response to interferon-gamma | 192 | 25 | 3.32E-11 | 9.40E-09 | PRKCD; TRIM21; OAS1; IRF1; IRF7; OAS3; TLR3; GCH1; MT2A; WNT5A; GBP1; OASL; OAS2; IFI30; IFITM1; ICAM1; JAK2; SOCS3; DAPK1; SOCS1; LGALS9; GBP4; STXBP2; IFITM2; CYP27B1 |
| GO:0098542 | defense response to other organism | 473 | 39 | 1.91E-10 | 4.06E-08 | PMAIP1; HIST1H2BD; PRKCD; IFI27; IFI6; BATF2; OAS1; SAMHD1; IRF1; IRF7; OAS3; TLR3; MX1; IFIT3; GBP1; OASL; OAS2; IFIT5; ISG15; IFITM1; IFIH1; DHX58; DDX58; MX2; STAT2; RSAD2; IFI44L; IFIT1; CXCL10; RTP4; ADGRB1; NT5C3A; BCL3; IFIT2; NOD2; UNC93B1; ISG20; GBP3; IFITM2 |
| GO:0048285 | organelle fission | 459 | 32 | 4.34E-07 | 6.34E-05 | MKI67; NCAPH; PTTG1; SGO1; CCNA2; TOP2A; CDC25C; NCAPG; CEP55; KIF14; DLGAP5; AURKB; MX1; ESPL1; WNT5A; CCNB1; ASPM; KIF2C; NUF2; CDCA8; ANLN; NEK2; MX2; STAT2; CYP26B1; MSX1; GPR3; OBSL1; MYBL2; KIF18B; PLK1; LIF |
| GO:0035455 | response to interferon-alpha | 20 | 7 | 4.48E-07 | 6.34E-05 | LAMP3; IFIT3; IFITM1; MX2; IFIT2; GATA3; IFITM2 |
| GO:0071900 | regulation of protein serine/threonine kinase activity | 497 | 32 | 2.44E-06 | 2.96E-04 | FZD5; PRKCD; CCNA2; CDC25C; TRIB1; MLKL; TRIB3; SPRY4; THBS1; IQGAP3; MAP4K1; DUSP6; FGF1; WNT5A; CCNB1; DUSP4; RGS3; DUSP5; HGF; SPRY2; CD74; DUSP2; JAK2; SERTAD1; PTPRJ; GADD45B; CEBPA; FZD10; PLK1; NOD2; RET; TPD52L1 |
| GO:0001818 | negative regulation of cytokine production | 256 | 21 | 3.69E-06 | 3.70E-04 | ZFP36; THBS1; IDO1; GBP1; ISG15; IFIH1; DHX58; ANGPT1; DDX58; CEACAM1; HGF; PDCD1LG2; CARD16; FN1; BCL3; CD274; LGALS9; GATA3; LAG3; INHBA; KLF2 |
| GO:0007162 | negative regulation of cell adhesion | 257 | 21 | 3.92E-06 | 3.70E-04 | PRKCD; IRF1; THBS1; IDO1; GBP1; TNC; ANGPT1; CEACAM1; PDCD1LG2; CD74; JAK2; SOCS1; PHLDB2; CD274; LGALS9; ZNF703; TRPV4; LAG3; RIPOR2; ARHGAP6; SPINT2 |
| GO:0002683 | negative regulation of immune system process | 416 | 28 | 4.59E-06 | 3.90E-04 | HES1; ZFP36; COL3A1; SAMHD1; LDLR; TRIB1; IRF1; TLR3; THBS1; IDO1; GBP1; DHX58; ANGPT1; CEACAM1; MYC; PDCD1LG2; SERPING1; CD74; IFIT1; PTPRJ; SOCS1; CD274; LGALS9; TMEM178A; CR1; LAG3; INHBA; RIPOR2 |

B)

| **GO symbol** | **Molecular Function** | **Size** | **Overlap** | **pValue** | **FDR** | **Genes** |
| --- | --- | --- | --- | --- | --- | --- |
| GO:0019900 | kinase binding | 711 | 37 | 1.15E-06 | 0.002150042 | FZD5; ZFP36; SGO1; PRKCD; CCNA2; RAC3; TOP2A; CDC25C; FAS; EEF1A2; TRIB1; MLKL; TRIB3; KIF14; GCH1; AURKB; CCNB1; ASPM; KIF20A; WARS; ANGPT1; CEACAM1; BCL2L14; RASGRP3; SPRY2; DUSP2; CARD16; JAK2; DSP; PTPRJ; SOCS1; CEBPA; RHOU; PLK1; NOD2; TRPV4; BRSK2 |
| GO:0042802 | identical protein binding | 1696 | 65 | 5.99E-06 | 0.005323588 | HES1; MCL1; HIST1H2BD; E2F8; PTGS2; TRIM9; TOP2A; IFI27; JAM3; PIK3AP1; IER5; TRIM21; FAS; CSF1; HJURP; SAMHD1; LDLR; TESC; TLR3; GCH1; THBS1; MX1; FGFR3; IFIT3; HSPB6; GBP1; SHANK1; MAOB; ACTN2; TAP1; HOXA1; ATF3; TNFSF18; IFIH1; ANXA9; WARS; BIRC5; DDX58; APLP1; CEACAM1; STAT2; TYMP; HGF; TNFSF10; GIPC2; MRAP2; CD74; ADA2; CARD16; JAK2; DAPK1; FN1; PGF; CEBPA; GPR50; NCAM2; PLK1; GBP4; HCN2; TPD52L1; TRPV4; GBP3; HAS3; INHBA; RIPOR2 |
| GO:0005102 | signaling receptor binding | 1538 | 60 | 8.51E-06 | 0.005323588 | COL3A1; JAG2; TSPOAP1; JAM3; CSF1; NCOA7; ADM; CHGB; THBS1; LGI1; FGF1; WNT5A; CCNB1; SHANK1; ACTN2; OASL; TNFSF13B; TAP1; NXPH3; TNFSF18; ADM2; ANGPT1; APLP1; TYMP; HGF; TNFSF10; MRAP2; CD74; ICAM1; ADA2; CXCL10; ETS2; RTP4; JAK2; PTPRJ; IL18BP; CHAC1; SOCS1; SECTM1; FN1; PGF; CTHRC1; LAMA5; RELN; PXDN; TAP2; GATA3; RGMA; SYTL3; UNC93B1; RNF43; LRRTM2; GRIN1; ARRDC3; LIF; CXCL11; CPE; LAG3; INHBA; ARTN |
| GO:0098772 | molecular function regulator | 1844 | 68 | 1.28E-05 | 0.005986595 | PPP1R1C; NCAPH; PTTG1; JAG2; PRKCD; CCNA2; ARHGAP26; SPTBN4; PPP1R3C; CSF1; TESC; TRIB1; TRIB3; DOCK10; ADM; CHGB; FGFR3; FGF1; WNT5A; CCNB1; ARHGAP25; DENND1C; ACTN2; TNFSF13B; SPTB; ARFGEF3; CISH; TNFSF18; DEPDC1; WARS; ADM2; RGS3; BIRC5; ANGPT1; TYMP; RASGRP3; HGF; TNFSF10; MCF2; SPRY2; TAGAP; SERPING1; MRAP2; CST1; ADA2; CXCL10; CARD16; JAK2; SOCS3; IL18BP; SOCS1; SECTM1; FN1; PGF; RASGEF1B; RGS11; PXDN; PLEKHG1; RET; LXN; GRIN1; BRSK2; LIF; CXCL11; INHBA; ARHGAP6; ARTN; SPINT2 |
| GO:0019901 | protein kinase binding | 631 | 31 | 2.71E-05 | 0.01015923 | FZD5; ZFP36; PRKCD; CCNA2; RAC3; TOP2A; CDC25C; EEF1A2; TRIB1; MLKL; TRIB3; KIF14; GCH1; CCNB1; ASPM; KIF20A; WARS; ANGPT1; CEACAM1; BCL2L14; SPRY2; DUSP2; JAK2; DSP; PTPRJ; SOCS1; RHOU; PLK1; NOD2; TRPV4; BRSK2 |
| GO:0032555 | purine ribonucleotide binding | 1850 | 66 | 4.94E-05 | 0.014822483 | MKI67; TUBB4A; RHEBL1; PBK; PRKCD; KIF5A; RAC3; TOP2A; EEF1A2; OAS1; SAMHD1; PLK3; ATP10A; TRIB1; MAST1; MLKL; TRIB3; KIF14; OAS3; PLK2; BVES; GCH1; AURKB; MX1; MAP4K1; FGFR3; KIF2C; SLFN12L; GBP1; KIF15; OASL; TAP1; OAS2; MYO3A; CMPK2; NEK2; KIF20A; IFIH1; WARS; DHX58; DDX58; MX2; IFI44L; DDX60L; RRAD; GUCY1A2; JAK2; DAPK1; HELZ2; GBP7; KIF18B; TAP2; TTLL6; RHOU; PLK1; GBP4; NOD2; RET; HCN2; TRPV4; CACNA1B; GBP3; BRSK2; DNAH10; TGM2; CHST15 |
| GO:0017076 | purine nucleotide binding | 1865 | 66 | 6.32E-05 | 0.014822483 | MKI67; TUBB4A; RHEBL1; PBK; PRKCD; KIF5A; RAC3; TOP2A; EEF1A2; OAS1; SAMHD1; PLK3; ATP10A; TRIB1; MAST1; MLKL; TRIB3; KIF14; OAS3; PLK2; BVES; GCH1; AURKB; MX1; MAP4K1; FGFR3; KIF2C; SLFN12L; GBP1; KIF15; OASL; TAP1; OAS2; MYO3A; CMPK2; NEK2; KIF20A; IFIH1; WARS; DHX58; DDX58; MX2; IFI44L; DDX60L; RRAD; GUCY1A2; JAK2; DAPK1; HELZ2; GBP7; KIF18B; TAP2; TTLL6; RHOU; PLK1; GBP4; NOD2; RET; HCN2; TRPV4; CACNA1B; GBP3; BRSK2; DNAH10; TGM2; CHST15 |
| GO:0032553 | ribonucleotide binding | 1865 | 66 | 6.32E-05 | 0.014822483 | MKI67; TUBB4A; RHEBL1; PBK; PRKCD; KIF5A; RAC3; TOP2A; EEF1A2; OAS1; SAMHD1; PLK3; ATP10A; TRIB1; MAST1; MLKL; TRIB3; KIF14; OAS3; PLK2; BVES; GCH1; AURKB; MX1; MAP4K1; FGFR3; KIF2C; SLFN12L; GBP1; KIF15; OASL; TAP1; OAS2; MYO3A; CMPK2; NEK2; KIF20A; IFIH1; WARS; DHX58; DDX58; MX2; IFI44L; DDX60L; RRAD; GUCY1A2; JAK2; DAPK1; HELZ2; GBP7; KIF18B; TAP2; TTLL6; RHOU; PLK1; GBP4; NOD2; RET; HCN2; TRPV4; CACNA1B; GBP3; BRSK2; DNAH10; TGM2; CHST15 |
| GO:0035639 | purine ribonucleoside triphosphate binding | 1786 | 63 | 1.05E-04 | 0.021895603 | MKI67; TUBB4A; RHEBL1; PBK; PRKCD; KIF5A; RAC3; TOP2A; EEF1A2; OAS1; SAMHD1; PLK3; ATP10A; TRIB1; MAST1; MLKL; TRIB3; KIF14; OAS3; PLK2; GCH1; AURKB; MX1; MAP4K1; FGFR3; KIF2C; SLFN12L; GBP1; KIF15; OASL; TAP1; OAS2; MYO3A; CMPK2; NEK2; KIF20A; IFIH1; WARS; DHX58; DDX58; MX2; IFI44L; DDX60L; RRAD; GUCY1A2; JAK2; DAPK1; HELZ2; GBP7; KIF18B; TAP2; TTLL6; RHOU; PLK1; GBP4; NOD2; RET; TRPV4; CACNA1B; GBP3; BRSK2; DNAH10; TGM2 |
| GO:0005085 | guanyl-nucleotide exchange factor activity | 329 | 19 | 1.33E-04 | 0.024083437 | SPTBN4; DOCK10; FGFR3; FGF1; DENND1C; ACTN2; SPTB; ARFGEF3; ANGPT1; RASGRP3; HGF; MCF2; TAGAP; JAK2; RASGEF1B; PLEKHG1; RET; GRIN1; ARTN |

Table S2. Results of differential expression analysis of imatinib-treated GIST (n=3) and non-treated GIST control samples (n=3)

| Gene id | *p* value | FC imatinib vs control |
| --- | --- | --- |
| CNN1 | 4.6E-06 | 9.728656045 |
| LRRTM1 | 2.3E-05 | 7.142608684 |
| OBSL1 | 0.010635 | 4.775416532 |
| HAS3 | 0.029413 | 4.639752981 |
| TSPOAP1 | 0.000539 | 4.304591989 |
| GALR2 | 0.001222 | 4.247789136 |
| PLCXD3 | 0.019136 | 4.015309601 |
| MAOB | 0.002346 | 3.67152772 |
| FMO3 | 0.035693 | 3.552486894 |
| LINC01297 | 0.043291 | 3.411367326 |
| ADA2 | 0.007589 | 3.410573903 |
| BEX1 | 0.017719 | 3.398823489 |
| TNFAIP8L3 | 0.013052 | 3.398160802 |
| PLCB2 | 4.24E-05 | 3.389555382 |
| PLPPR4 | 0.000876 | 3.307093475 |
| MPPED2 | 0.001137 | 3.293783881 |
| NOVA1 | 0.046308 | 3.200312191 |
| PLEKHA6 | 0.008875 | 3.194495641 |
| HRASLS | 0.023769 | 3.034893231 |
| SPARCL1 | 0.030592 | 3.017681673 |
| CHGB | 0.00138 | 3.000890656 |
| DENND1C | 0.002445 | 2.985812458 |
| TMEM47 | 0.011418 | 2.965589812 |
| TUBB4A | 0.000244 | 2.950400334 |
| HCN2 | 0.018858 | 2.926074273 |
| LRAT | 0.037193 | 2.908156754 |
| EVPL | 3.07E-05 | 2.906474472 |
| TPSG1 | 0.005189 | 2.876142958 |
| C10orf111 | 0.04382 | 2.85117135 |
| ANXA9 | 0.003449 | 2.795420679 |
| RDH10-AS1 | 0.000597 | 2.782274846 |
| HSPB6 | 0.001922 | 2.744235939 |
| GUCY1A2 | 0.006813 | 2.739361118 |
| LURAP1 | 0.002302 | 2.739082745 |
| NKX6-1 | 0.00015 | 2.731319311 |
| SLITRK5 | 0.006035 | 2.71399512 |
| HSF4 | 0.000208 | 2.712590751 |
| MB21D2 | 0.001524 | 2.701929071 |
| KCNT1 | 0.009252 | 2.681421149 |
| RGS11 | 0.012825 | 2.660085125 |
| KIF5A | 0.000459 | 2.650852639 |
| PART1 | 0.035299 | 2.627773701 |
| PGF | 0.010256 | 2.59665346 |
| FAM156B | 0.016812 | 2.573725306 |
| SPINT2 | 0.046971 | 2.565467661 |
| MRAP2 | 0.006385 | 2.55636137 |
| HAGHL | 0.005582 | 2.52432736 |
| IGFBP5 | 0.009102 | 2.522007264 |
| SMCO3 | 0.002713 | 2.519292893 |
| RRAD | 0.006548 | 2.509600387 |
| AC004069.1 | 0.003673 | 2.500422161 |
| JPH4 | 0.017993 | 2.499791215 |
| JAM3 | 0.000569 | 2.498591316 |
| NAP1L3 | 0.013738 | 2.479738035 |
| CACNA1B | 0.022044 | 2.47535405 |
| PTGDS | 0.002524 | 2.43734775 |
| MCAM | 0.023347 | 2.436063269 |
| CILP2 | 0.004562 | 2.429911178 |
| CORO6 | 9.73E-05 | 2.4241782 |
| JAG2 | 0.000416 | 2.42256359 |
| TMEM178A | 0.019998 | 2.414143759 |
| HS6ST2 | 0.008987 | 2.407176494 |
| EEF1A1P25 | 0.014457 | 2.392127182 |
| CBY3 | 0.006456 | 2.382744534 |
| SMIM34A | 0.004947 | 2.376847527 |
| CALY | 0.04842 | 2.366845281 |
| AC010343.1 | 0.026059 | 2.36362388 |
| RPL10AP6 | 0.012683 | 2.361054232 |
| CYP26B1 | 0.004984 | 2.356124923 |
| RPL5P30 | 0.000474 | 2.351647601 |
| RGMA | 0.015247 | 2.345028668 |
| GALNT9 | 0.007004 | 2.327165634 |
| IL17REL | 0.013702 | 2.326997184 |
| RSPH1 | 0.00327 | 2.321164244 |
| AL031847.1 | 0.002827 | 2.318993486 |
| ARHGAP6 | 0.04474 | 2.316299327 |
| AL512625.2 | 0.003432 | 2.311566868 |
| GOT1L1 | 0.019357 | 2.299886389 |
| SHISA9 | 0.006489 | 2.293583732 |
| CPE | 0.037932 | 2.29343123 |
| AC010907.1 | 0.01636 | 2.28878834 |
| CCDC78 | 0.007473 | 2.283979326 |
| MAP4K1 | 0.001821 | 2.283681889 |
| PPP1R3C | 0.000767 | 2.280506296 |
| TEX45 | 0.000216 | 2.267292029 |
| SLC16A11 | 0.000318 | 2.263006913 |
| MYL9 | 0.000712 | 2.258407541 |
| TRIM9 | 0.000293 | 2.246213767 |
| AC003071.1 | 0.021478 | 2.243688893 |
| AC020779.1 | 0.027626 | 2.236395676 |
| SPTBN4 | 0.000665 | 2.235916801 |
| SUGCT | 0.030466 | 2.235415918 |
| ADAMTS9-AS2 | 0.041055 | 2.23312431 |
| AC011484.1 | 0.014193 | 2.232058743 |
| C16orf96 | 0.01223 | 2.22810177 |
| INHBA | 0.041025 | 2.22215261 |
| SHANK1 | 0.002181 | 2.221327636 |
| CCDC17 | 0.00601 | 2.220168181 |
| SUPT4H1P2 | 0.018999 | 2.20930608 |
| NAT8L | 0.005069 | 2.209185483 |
| APLP1 | 0.00412 | 2.202165207 |
| ARFGEF3 | 0.002933 | 2.192064337 |
| PIK3CD-AS2 | 0.006127 | 2.189594291 |
| MT-TR | 0.048205 | 2.177426147 |
| CPNE7 | 0.012232 | 2.176217881 |
| MEX3B | 0.000399 | 2.176149877 |
| AL139349.1 | 0.007604 | 2.175866516 |
| EWSAT1 | 0.009509 | 2.175335738 |
| ESRP2 | 0.000294 | 2.175217243 |
| MT1A | 0.000823 | 2.170127679 |
| TAGLN | 0.007971 | 2.167184279 |
| DDIT4L | 0.002869 | 2.162378065 |
| LTBP1 | 0.014041 | 2.162129781 |
| SMIM10L2B | 0.012845 | 2.161121042 |
| RPS15P5 | 0.033251 | 2.158089264 |
| C16orf45 | 0.018467 | 2.155668473 |
| CD48 | 0.013691 | 2.154428277 |
| WASIR2 | 0.011917 | 2.150297046 |
| LMCD1-AS1 | 0.000512 | 2.149627797 |
| MAST1 | 0.001126 | 2.143701766 |
| LINC01234 | 0.042415 | 2.140751107 |
| STEAP3 | 8.63E-05 | 2.140671098 |
| MYLK-AS1 | 0.011401 | 2.137418523 |
| C16orf74 | 0.00044 | 2.136207583 |
| GNG4 | 0.023395 | 2.136088318 |
| SNX32 | 0.003125 | 2.125347966 |
| LHX6 | 0.016309 | 2.124144507 |
| AC004969.1 | 0.001181 | 2.122768511 |
| RAC3 | 0.0005 | 2.121796695 |
| LAMA5 | 0.010734 | 2.118746261 |
| NR1I2 | 0.000696 | 2.117032884 |
| AL451069.1 | 0.005081 | 2.109768454 |
| SARDH | 0.005561 | 2.108960789 |
| CA14 | 0.002907 | 2.104839551 |
| TPD52L1 | 0.018976 | 2.095384957 |
| GGTA1P | 0.005656 | 2.091733079 |
| CEBPA | 0.012448 | 2.090773726 |
| RPL14P1 | 0.003777 | 2.087697807 |
| FBXO32 | 0.012703 | 2.087523141 |
| FZD5 | 0.000152 | 2.085819914 |
| MST1P2 | 0.013409 | 2.083032049 |
| AC124944.2 | 0.034173 | 2.080110493 |
| USP2-AS1 | 0.008367 | 2.077699213 |
| ZNF703 | 0.014616 | 2.072859729 |
| AC110373.1 | 0.032826 | 2.072097563 |
| AC073133.2 | 0.015378 | 2.070036156 |
| NRADDP | 0.008936 | 2.067792076 |
| EEF1A2 | 0.000783 | 2.067290362 |
| STPG3 | 0.018492 | 2.051819755 |
| SNORD59A | 0.007178 | 2.048718451 |
| TESC | 0.001005 | 2.048190077 |
| FAM225B | 0.019841 | 2.047725418 |
| LINC02415 | 0.034819 | 2.047483004 |
| GRIN1 | 0.024096 | 2.032420286 |
| SLC9A3 | 0.001119 | 2.030698507 |
| BRSK2 | 0.026724 | 2.029102325 |
| CNTN1 | 0.003138 | 2.024214835 |
| TTYH2 | 0.005592 | 2.023037488 |
| MCF2 | 0.005203 | 2.016160948 |
| DLGAP2 | 0.020231 | 2.014966727 |
| DPY19L2P4 | 0.01993 | 2.011251292 |
| AC008443.5 | 0.044244 | 2.011188434 |
| AL592295.1 | 0.003198 | 2.010140745 |
| WDR97 | 0.008341 | 2.009334002 |
| AL355877.1 | 0.005744 | 2.006596265 |
| HBQ1 | 0.001809 | 2.002764195 |
| ANKRD13B | 0.002923 | 2.000422977 |
| NFKBIE | 0.005602 | 0.504940623 |
| AC096677.2 | 0.045263 | 0.504549085 |
| SAMHD1 | 0.00094 | 0.504367275 |
| DEPDC1 | 0.003514 | 0.504290139 |
| UGCG | 0.014541 | 0.50421701 |
| HGF | 0.005083 | 0.504048347 |
| AC116407.1 | 0.011993 | 0.503986071 |
| CDCA3 | 0.002742 | 0.503768097 |
| TOP2A | 0.0005 | 0.503508729 |
| CCNB1 | 0.001957 | 0.502748529 |
| TRPV4 | 0.020335 | 0.502590124 |
| GCH1 | 0.001509 | 0.502292508 |
| NOD2 | 0.015421 | 0.502278028 |
| PLK3 | 0.000992 | 0.502159799 |
| TAP2 | 0.013334 | 0.501966233 |
| PHLDB2 | 0.009346 | 0.501964425 |
| KIF15 | 0.002205 | 0.501840365 |
| NCAM2 | 0.014453 | 0.501389145 |
| FGFR3 | 0.001837 | 0.501316804 |
| PXDN | 0.013016 | 0.501084276 |
| JAK2 | 0.008606 | 0.500884322 |
| CFHR3 | 0.025224 | 0.500868517 |
| PPP1R1C | 0.000154 | 0.500095418 |
| SERTAD1 | 0.008831 | 0.499144136 |
| ENC1 | 0.017347 | 0.498662473 |
| HIST1H2AG | 0.020106 | 0.498440648 |
| TLR3 | 0.001373 | 0.497675871 |
| GRAP | 0.004706 | 0.496305175 |
| CMYA5 | 0.004917 | 0.496101365 |
| SGO1 | 0.000415 | 0.495862618 |
| CKAP2L | 0.000496 | 0.495538297 |
| ELK4 | 0.003681 | 0.49542753 |
| PIMREG | 0.011085 | 0.494957159 |
| AURKB | 0.001596 | 0.494571063 |
| BVES | 0.0015 | 0.494086373 |
| IFIT5 | 0.002907 | 0.493752696 |
| KIF18B | 0.013307 | 0.493254883 |
| HIST1H1C | 0.010403 | 0.493030712 |
| PTPRJ | 0.008831 | 0.492456949 |
| ANLN | 0.003027 | 0.492382394 |
| AC008663.2 | 0.02275 | 0.491769321 |
| ZNF816 | 0.005038 | 0.491724164 |
| LZTS1 | 0.014801 | 0.491223593 |
| LXN | 0.023132 | 0.490309642 |
| ATF3 | 0.002982 | 0.489973938 |
| HMMR | 0.003234 | 0.489818933 |
| STXBP2 | 0.023813 | 0.489628436 |
| MLKL | 0.001144 | 0.48944835 |
| RGS3 | 0.003932 | 0.489269512 |
| GBP3 | 0.025027 | 0.489114762 |
| GADD45B | 0.010589 | 0.488869268 |
| FAM46A | 0.002683 | 0.488734652 |
| IFI35 | 0.005622 | 0.486223799 |
| LGI4 | 0.016837 | 0.485325733 |
| CHAC1 | 0.009003 | 0.485270975 |
| ANGPT1 | 0.004013 | 0.484391242 |
| SPC24 | 0.001072 | 0.482514413 |
| TNFAIP2 | 0.018328 | 0.482300146 |
| CR1 | 0.021071 | 0.481879404 |
| IQGAP3 | 0.001768 | 0.48120189 |
| GPR50 | 0.014063 | 0.48046266 |
| TROAP | 0.007114 | 0.479140816 |
| AC109779.1 | 0.007194 | 0.478512567 |
| GTSE1 | 0.003286 | 0.478302884 |
| CD74 | 0.006535 | 0.477835779 |
| SLC8A1 | 0.038263 | 0.477747838 |
| NCAPG | 0.000809 | 0.477254078 |
| APOL1 | 0.001709 | 0.476983079 |
| RHOU | 0.013828 | 0.476532179 |
| LINC02006 | 0.009298 | 0.47581066 |
| TEKT2 | 0.043212 | 0.475760755 |
| KIAA0087 | 0.007592 | 0.475414099 |
| SPC25 | 0.001443 | 0.475371151 |
| PTTG1 | 0.000307 | 0.473761964 |
| ENPEP | 0.001295 | 0.472947305 |
| SLC6A9 | 0.004918 | 0.472484568 |
| PBK | 0.000352 | 0.471366905 |
| GIPC2 | 0.005292 | 0.470512717 |
| ARHGAP26 | 0.000531 | 0.469792009 |
| BTN2A3P | 0.022038 | 0.469533931 |
| ESPL1 | 0.001896 | 0.469371107 |
| KNL1 | 0.002441 | 0.469102466 |
| IER2 | 0.00023 | 0.468982578 |
| AC021086.1 | 0.043631 | 0.468409289 |
| TMEM92 | 0.012539 | 0.467929835 |
| RNF213 | 0.005458 | 0.467884718 |
| IFITM2 | 0.025899 | 0.46722836 |
| ADAMTS2 | 0.015458 | 0.465987835 |
| CARD16 | 0.008302 | 0.465265288 |
| NCAPH | 0.000279 | 0.465175722 |
| BIRC5 | 0.00396 | 0.465061727 |
| MLIP | 0.022475 | 0.464934992 |
| CDC25C | 0.000517 | 0.464460615 |
| PRKCD | 0.000418 | 0.46411259 |
| NEK2 | 0.003115 | 0.463549561 |
| PDZD2 | 0.001009 | 0.463234293 |
| PCDH18 | 0.016762 | 0.462576728 |
| NUF2 | 0.002677 | 0.462185474 |
| C1orf226 | 0.030744 | 0.462012476 |
| LAP3 | 0.004959 | 0.461030276 |
| MGAT3 | 0.043194 | 0.458969278 |
| LINC00968 | 0.001138 | 0.458393946 |
| P2RY8 | 0.021626 | 0.457266994 |
| PLEKHO2 | 0.001511 | 0.456807481 |
| TNFSF10 | 0.005099 | 0.456480994 |
| CDCA8 | 0.00294 | 0.456457087 |
| GDPD5 | 0.003894 | 0.455384783 |
| VWA3B | 0.000225 | 0.454353337 |
| SORCS2 | 0.013849 | 0.453502142 |
| NCOA7 | 0.000896 | 0.453206874 |
| CASZ1 | 0.011722 | 0.452992019 |
| GPR3 | 0.006323 | 0.452587082 |
| WNT5A | 0.00195 | 0.45011247 |
| SLC15A3 | 0.01552 | 0.449361253 |
| SP110 | 0.002063 | 0.448946143 |
| SLC44A3 | 0.008741 | 0.448257934 |
| MTTP | 0.009853 | 0.447740254 |
| PLA1A | 0.037436 | 0.447572693 |
| MYBL2 | 0.012463 | 0.446111166 |
| LYVE1 | 0.005939 | 0.444432531 |
| MYO3A | 0.002768 | 0.442760521 |
| COL3A1 | 0.000238 | 0.440202499 |
| DHX58 | 0.003827 | 0.439591254 |
| RELN | 0.012295 | 0.439525001 |
| RRM2 | 0.001352 | 0.439086505 |
| WARS | 0.003652 | 0.439064489 |
| KIF14 | 0.001231 | 0.438942533 |
| ASPM | 0.001987 | 0.438765632 |
| STAT2 | 0.004524 | 0.438722124 |
| TBX3 | 0.025924 | 0.438207828 |
| TNFRSF10A | 0.004454 | 0.437934203 |
| ZNFX1 | 0.003011 | 0.43789963 |
| P3H2 | 0.040308 | 0.437700707 |
| HIST1H2BC | 0.009515 | 0.436004501 |
| AL035681.1 | 0.003743 | 0.434878103 |
| TRIM21 | 0.000733 | 0.434420402 |
| ADGRB1 | 0.008695 | 0.434365359 |
| HJURP | 0.000927 | 0.434153053 |
| CENPA | 0.000698 | 0.431681331 |
| C2orf50 | 0.029145 | 0.430165849 |
| KLF2 | 0.042507 | 0.43000689 |
| AC073529.1 | 0.012832 | 0.429104453 |
| OSBPL10-AS1 | 0.008718 | 0.428915863 |
| SPTB | 0.00293 | 0.427885798 |
| KIF20A | 0.003126 | 0.42710116 |
| TRIB3 | 0.001194 | 0.427087067 |
| LINC00992 | 0.043111 | 0.426022882 |
| LGALS9 | 0.013499 | 0.424303193 |
| ARNTL2 | 0.015674 | 0.424042882 |
| RHEBL1 | 0.00034 | 0.423678037 |
| STX11 | 0.024178 | 0.423632183 |
| KCTD14 | 0.000859 | 0.422983562 |
| TRIM47 | 0.00781 | 0.422728398 |
| HLA-V | 0.021697 | 0.422460282 |
| ETS2 | 0.00788 | 0.422411415 |
| DUSP2 | 0.008106 | 0.421565875 |
| PLEKHG1 | 0.016365 | 0.420734452 |
| CLMN | 0.005526 | 0.420701139 |
| KIF2C | 0.001993 | 0.420341816 |
| MKI67 | 0.000207 | 0.4187197 |
| CEP55 | 0.000879 | 0.418508512 |
| HOXA1 | 0.002837 | 0.416591738 |
| EMP1 | 7.38E-05 | 0.41643632 |
| LHFPL6 | 0.019149 | 0.416065818 |
| PLK1 | 0.014649 | 0.415690075 |
| AL354702.1 | 0.006848 | 0.414630918 |
| FZD10 | 0.013556 | 0.411622361 |
| ACKR4 | 0.010003 | 0.41128314 |
| DOCK10 | 0.001268 | 0.409953633 |
| HELZ2 | 0.011139 | 0.40797123 |
| TRIB1 | 0.001101 | 0.407674023 |
| UGT3A2 | 0.012505 | 0.407587089 |
| CCNA2 | 0.000419 | 0.407077474 |
| NXPH3 | 0.003186 | 0.406820642 |
| GBP4 | 0.014929 | 0.406244514 |
| DNAH10 | 0.034129 | 0.406223999 |
| LGI1 | 0.001748 | 0.404946854 |
| MX1 | 0.00168 | 0.404086684 |
| FN1 | 0.00945 | 0.403924602 |
| AC139103.1 | 0.041884 | 0.403341688 |
| BCL3 | 0.009787 | 0.403107886 |
| LINC00920 | 0.04265 | 0.402212324 |
| BLZF1 | 0.008113 | 0.402152449 |
| FCRL5 | 0.010842 | 0.401623616 |
| ETV7 | 0.007969 | 0.401162194 |
| LCMT2 | 0.002191 | 0.400225574 |
| SLC7A11 | 0.022712 | 0.397586394 |
| AC093110.1 | 0.000877 | 0.397452529 |
| E2F8 | 0.000269 | 0.394772646 |
| HERC6 | 0.000419 | 0.394692813 |
| FAS | 0.000737 | 0.393045315 |
| WDR49 | 0.001091 | 0.391677786 |
| HIST1H2AC | 0.000296 | 0.391291951 |
| SLC37A1 | 0.006626 | 0.389756917 |
| TYMP | 0.004764 | 0.389620329 |
| XAF1 | 0.003006 | 0.389558079 |
| RTP4 | 0.008058 | 0.388908653 |
| ARTN | 0.046404 | 0.386766037 |
| CILP | 0.032564 | 0.386664448 |
| RARRES3 | 0.005407 | 0.386419057 |
| DDX60L | 0.006007 | 0.385868334 |
| ASNSP1 | 0.028745 | 0.385577411 |
| IFI44 | 0.019685 | 0.384134391 |
| RBM47 | 0.000339 | 0.381585924 |
| SPAG6 | 0.034302 | 0.381281032 |
| UNC5C | 0.005001 | 0.37912893 |
| CSRNP1 | 0.000306 | 0.377970079 |
| MX2 | 0.004352 | 0.376217792 |
| LRRN1 | 0.028608 | 0.376064841 |
| RNF43 | 0.019766 | 0.376043189 |
| ADGRE5 | 0.001919 | 0.375686416 |
| SLC52A1 | 0.013284 | 0.373846189 |
| MSX1 | 0.006217 | 0.372551346 |
| PDCD1LG2 | 0.00536 | 0.372407784 |
| PLEKHF1 | 0.00735 | 0.372298935 |
| HCAR2 | 0.011149 | 0.372274436 |
| GBP7 | 0.012577 | 0.372160498 |
| IFI44L | 0.005359 | 0.370557871 |
| CHST15 | 0.045121 | 0.368814471 |
| NT5C3A | 0.008994 | 0.36876894 |
| TAP1 | 0.002613 | 0.368444807 |
| CH25H | 0.010393 | 0.368300496 |
| AC078788.1 | 0.012884 | 0.36766623 |
| TLDC2 | 0.020559 | 0.367294686 |
| CRYBG1 | 0.0124 | 0.367044874 |
| MCL1 | 0.000146 | 0.36578871 |
| MAFF | 0.00172 | 0.365765705 |
| IER5 | 0.000728 | 0.365688125 |
| VNN2 | 0.009976 | 0.365360498 |
| PIK3AP1 | 0.000727 | 0.365259455 |
| RNF144B | 0.000531 | 0.365163208 |
| CEBPD | 0.000765 | 0.362098775 |
| DLGAP5 | 0.001591 | 0.359571032 |
| SYTL3 | 0.015823 | 0.359394246 |
| RET | 0.017188 | 0.357493418 |
| IFITM1 | 0.003295 | 0.357221975 |
| CCDC146 | 0.000572 | 0.356468169 |
| CFI | 0.002228 | 0.356340002 |
| SAMD9L | 0.0061 | 0.354836488 |
| MIR3142HG | 0.001042 | 0.349396519 |
| SERPING1 | 0.006203 | 0.348922799 |
| ARHGAP25 | 0.002188 | 0.346297903 |
| FCRL1 | 0.028848 | 0.345863171 |
| IGDCC3 | 0.002371 | 0.345238358 |
| SLC9C1 | 0.000472 | 0.344629069 |
| FOSL2 | 0.000376 | 0.343901509 |
| RASGRP3 | 0.004986 | 0.341960456 |
| CSF1 | 0.000806 | 0.341641069 |
| AC096533.1 | 0.003154 | 0.340645269 |
| MT2A | 0.001581 | 0.340202788 |
| UNC93B1 | 0.016381 | 0.340119522 |
| AP000808.1 | 0.001567 | 0.340100838 |
| EPSTI1 | 0.002863 | 0.339692543 |
| IL18BP | 0.008976 | 0.338777657 |
| LIF | 0.030125 | 0.33847191 |
| CD274 | 0.010062 | 0.337567883 |
| GATA3 | 0.015142 | 0.337409493 |
| LRRTM2 | 0.019911 | 0.334866524 |
| DDX58 | 0.004104 | 0.333727741 |
| HIST1H2BD | 0.000175 | 0.332867762 |
| TMEM2 | 0.000971 | 0.332837663 |
| SLFN12L | 0.002065 | 0.332818496 |
| CYP27B1 | 0.033058 | 0.332525847 |
| RBPMS-AS1 | 0.002731 | 0.330793315 |
| FGF1 | 0.001931 | 0.326380606 |
| CTGF | 0.000576 | 0.325480791 |
| LINC00364 | 0.005057 | 0.323064075 |
| SOCS3 | 0.008725 | 0.322411279 |
| SLC4A4 | 0.009987 | 0.321358667 |
| PCDH17 | 0.000129 | 0.320935925 |
| OAS3 | 0.001344 | 0.320651987 |
| ADM2 | 0.003782 | 0.320065361 |
| PLK2 | 0.00145 | 0.318694724 |
| SEPT4 | 0.007397 | 0.316707992 |
| AL031599.1 | 0.008772 | 0.313699074 |
| IFI30 | 0.002832 | 0.310198042 |
| ODF3B | 0.00883 | 0.310126417 |
| CXCR2P1 | 0.049205 | 0.308556021 |
| PHLDA1 | 0.00086 | 0.306596112 |
| IFIH1 | 0.003306 | 0.305089352 |
| ADAMTS1 | 0.003102 | 0.304720407 |
| ZBTB42 | 0.005988 | 0.302320563 |
| RAMP3 | 0.001002 | 0.301864064 |
| PTGS2 | 0.000277 | 0.300632003 |
| TNC | 0.002589 | 0.299977292 |
| IFI27 | 0.000526 | 0.299681847 |
| CD5L | 0.003776 | 0.296846386 |
| DAPK1 | 0.008967 | 0.294238083 |
| SECTM1 | 0.009059 | 0.293040986 |
| LDLR | 0.000968 | 0.290763956 |
| LAG3 | 0.040163 | 0.289830198 |
| MYC | 0.004223 | 0.28795199 |
| TAGAP | 0.005427 | 0.287859268 |
| HCAR3 | 0.01538 | 0.284207916 |
| SPRY2 | 0.005239 | 0.283171504 |
| IRF7 | 0.001338 | 0.281203017 |
| FRMD3 | 0.006403 | 0.280268042 |
| HAPLN3 | 0.010629 | 0.27925045 |
| GBP1P1 | 0.007036 | 0.278427358 |
| ARRDC3 | 0.026034 | 0.277024188 |
| IFIT3 | 0.001847 | 0.275630747 |
| MS4A14 | 0.009098 | 0.274097646 |
| SLCO4C1 | 0.040189 | 0.273979226 |
| DSP | 0.00877 | 0.272131914 |
| OAS1 | 0.000909 | 0.270299706 |
| IRF1 | 0.001178 | 0.270117559 |
| RIPOR2 | 0.044465 | 0.268875464 |
| RSAD2 | 0.004596 | 0.268756337 |
| NRIR | 0.024496 | 0.268755391 |
| HCAR1 | 0.005195 | 0.264826508 |
| IFIT2 | 0.011549 | 0.261820184 |
| GBP1 | 0.002082 | 0.26006031 |
| USP18 | 0.001776 | 0.257687128 |
| ADM | 0.001309 | 0.256337506 |
| CMPK2 | 0.002779 | 0.254198186 |
| ACTN2 | 0.002457 | 0.250593558 |
| OAS2 | 0.002714 | 0.248136843 |
| CISH | 0.003074 | 0.246580722 |
| SAMD9 | 0.00846 | 0.246384628 |
| RUFY4 | 0.026293 | 0.23992191 |
| JUNB | 6.47E-05 | 0.225331787 |
| PMAIP1 | 5E-05 | 0.225061418 |
| HES1 | 7.09E-05 | 0.224848837 |
| ISG15 | 0.00293 | 0.222941891 |
| SOCS1 | 0.009044 | 0.222934279 |
| MS4A7 | 0.001081 | 0.22071744 |
| CEACAM1 | 0.004211 | 0.219762289 |
| LRRC4 | 0.004762 | 0.216690649 |
| CTHRC1 | 0.010368 | 0.216073103 |
| GRIP2 | 0.002313 | 0.215617506 |
| BCL2L14 | 0.004867 | 0.214214184 |
| ZBP1 | 0.004508 | 0.209539964 |
| SPRY4 | 0.001418 | 0.203559316 |
| IFI6 | 0.000571 | 0.203303758 |
| RASGEF1B | 0.011399 | 0.202953558 |
| IDO1 | 0.002 | 0.201298072 |
| TNFSF13B | 0.002586 | 0.19450526 |
| GMPR | 0.00571 | 0.191429954 |
| TGM2 | 0.038357 | 0.187612334 |
| ICAM1 | 0.007379 | 0.184452461 |
| IFIT1 | 0.006874 | 0.176974116 |
| BATF2 | 0.000782 | 0.171323977 |
| AL138900.2 | 0.000169 | 0.166605873 |
| TTLL6 | 0.013644 | 0.163149968 |
| FOS | 6.07E-07 | 0.162162327 |
| CYR61 | 8.3E-05 | 0.149794548 |
| LAMP3 | 0.000145 | 0.147919057 |
| AC025470.2 | 0.006319 | 0.147271352 |
| KCNC1 | 0.024417 | 0.140210653 |
| TNFSF18 | 0.003246 | 0.138442986 |
| ZFP36 | 0.000216 | 0.133602759 |
| PSG4 | 0.002629 | 0.130586053 |
| SLC6A19 | 0.001767 | 0.127654597 |
| ISG20 | 0.017674 | 0.121439462 |
| OASL | 0.00247 | 0.121281106 |
| CXCL11 | 0.032393 | 0.107797839 |
| ATP10A | 0.001013 | 0.104724892 |
| DUSP5 | 0.004453 | 0.090815648 |
| DUSP4 | 0.003298 | 0.089414431 |
| THBS1 | 0.001549 | 0.068447063 |
| CXCL10 | 0.007617 | 0.059439512 |
| DUSP6 | 0.001916 | 0.055822533 |
| EGR1 | 0.000166 | 0.043960337 |
| CST1 | 0.006999 | 0.03912696 |
